# Lipidomics in Brain Health and Neurodegenerative Diseases: Mechanisms, Biomarkers, and Therapeutic Perspectives

**DOI:** 10.3390/diagnostics16213589

**Published:** 2026-10-30

**Authors:** Arshad Husain Rahmani, Tarique Sarwar

**Affiliations:** Department of Medical Laboratories, College of Applied Medical Sciences, Qassim University, Buraydah 51452, Saudi Arabia

**Keywords:** lipidomics, neurodegeneration, Alzheimer’s disease, Parkinson’s disease, biomarkers, lipid metabolism, precision medicine, neuroinflammation

## Abstract

Lipids constitute nearly half of the brain’s dry weight and play a crucial role in maintaining the function of the central nervous system (CNS). Beyond their well-known function as structural components of cellular membranes and energy storage, lipids are now identified as dynamic bioactive mediators that regulate neuronal signaling, synaptic plasticity, membrane integrity and neuroinflammatory responses. Emerging evidence demonstrates that altered lipid homeostasis is associated with the pathogenesis of neurodegenerative diseases, including Alzheimer’s disease (AD), Parkinson’s disease (PD), amyotrophic lateral sclerosis (ALS) and Huntington’s disease (HD). In this review, we highlight key pathophysiological mechanisms, including dysregulation of lipid transport by cholesterol and apolipoprotein E4 (ApoE4), which may influence the aggregation of amyloid-β in AD. We discuss sphingolipid dysregulation and impaired glucocerebrosidase activity in modulating α-synuclein pathology in PD. Furthermore, we emphasize the importance of polyunsaturated fatty acids (PUFAs) and their oxidized forms in the regulation of oxidative stress, ferroptosis, and neuronal cell death in ALS and HD. Besides mechanistic information, this review also highlights the recent advancements in modern lipidomic technologies, such as high-resolution mass spectrometry and multi-omics integration, which have facilitated the comprehensive characterization of disease-associated lipid alterations. Emerging lipid signatures, including alterations in ceramide, cholesterol metabolism and lipid droplet biogenesis in glial cells, are being investigated as candidate biomarkers and therapeutic targets. Collectively, we outline a strategy for exploiting the brain lipidome to explore the potential for personalized disease-modifying strategies against neurodegeneration by integrating mechanistic, diagnostic and therapeutic insights.

## 1. Introduction

Lipid metabolism is a fundamental process for maintaining brain homeostasis and contributes to energy balance, membrane structure, and signaling pathways that are essential for neuronal and glial function. Lipids are highly structurally diverse, due to different lengths of acyl chains, degrees of saturation, and head group modifications, all of which are necessary for their diverse functions within the CNS [1]. These molecules are crucial in maintaining the integrity of cellular membranes, signaling pathways and energy storage, as well as acting as precursors to neuroactive mediators [2]. Disruption of lipid homeostasis is therefore emerging as a key mechanism that is responsible for the progression of neurodegenerative diseases, such as AD and PD [3,4]. Lipid metabolic dysfunction further disrupts intracellular signaling, membrane fluidity, and may promote protein aggregation, thereby leading to neuronal impairment [5].

Brain aging is a natural process that is associated with substantial changes in diverse lipid species and increased susceptibility to age-related neurodegenerative disorders [6]. Changes in lipids impact many different classes of lipids, including fatty acyls, glycerolipids, phospholipids, sphingolipids, and sterols, all of which have specific roles in neuronal maintenance, cell signaling and membrane dynamics [7]. Interestingly, the dysregulation of lipid metabolism has been associated with cognitive impairments observed in various disorders such as autism, schizophrenia, etc. [8]. These have made lipidomics an emerging high-throughput analytical platform to understand the complex relationship between lipid metabolism and neurodegenerative disorders [9]. This advanced analytical method allows the identification of disease-specific lipid biomarkers which might be useful for early diagnosis and disease progression, thus improving the understanding of the mechanism of neurodegenerative diseases [9,10]. The disruption of lipid metabolism may have substantial effects on brain health and functions [11,12]. With aging, these changes in lipid regulation lead to neurodegeneration, such as lipid exchange dysfunction between glia and neurons, and increased levels of lipid-peroxidation products resulting in oxidative stress and neuroinflammatory damage [13,14].

This review aims to provide a summary of the recent understanding of the mechanistic role of lipids in brain health and neurodegeneration, focusing on emerging lipidomic biomarkers and therapeutic approaches targeting lipid metabolic pathways. Lipid dysregulation, with ceramide accumulation, defects in cholesterol esterification, and oxidized lipid-induced neuroinflammation, is an important factor in the regulation of microglia, which plays a pivotal role in the pathogenesis of neurodegenerative disease [15]. Such dysregulations can be evident as distinct alterations in phospholipid species, such as increased levels of phospholipid hydroxides derived from arachidonic acid and changes in the azelaoyl phospholipid, between memory-impaired and unimpaired cognitive function [16]. In this review, we combine four complementary aspects of lipid biology in neurodegeneration: (i) mechanistic alterations of lipid metabolism and membrane homeostasis, (ii) disease-associated lipidomic signatures, (iii) analytical and clinical considerations for developing lipid-based biomarkers, and (iv) emerging therapeutic approaches targeting lipid metabolism or lipid-membrane interaction. By integrating mechanistic evidence with lipidomic discovery and validation in a translational context, we aim to provide a framework to assess the contribution of lipid alterations to disease mechanisms, as candidate biomarkers and as a potential therapeutic target. Although previous reviews have discussed lipid dysregulation and lipidomic biomarkers in neurodegenerative diseases, an important challenge is to connect mechanistic findings with biomarker development and the clinical translation of lipid-directed interventions. The present narrative review addresses this gap by integrating four interconnected areas of lipid biology: cholesterol and apolipoprotein E (APOE), sphingolipids, polyunsaturated fatty acids (PUFAs), and lipid droplets. These areas are considered across AD, PD, HD, and ALS, with emphasis on disease-specific mechanisms, analytical lipidomics, biofluid selection, diagnostic validation, and the clinical status of therapeutic candidates. A further focus is the critical distinction between associations observed in human studies, mechanistic findings from cellular and animal models, and outcomes from clinical trials. Rather than assuming that the same lipid pathways are causal across all four diseases, this review uses the four-area framework to organize overlapping mechanisms while highlighting disease-specific differences, methodological limitations, and translational gaps.

### Literature Search Strategy

This narrative review examines the role of lipid metabolism in brain health and its involvement in AD, PD, HD, and ALS, with particular emphasis on cholesterol and APOE, sphingolipids, PUFAs and lipid peroxidation, and lipid droplets. The review was conducted through searches of PubMed, Scopus, and Google Scholar with a focus on publications from 2007 to 2026 using the combinations of terms related to lipid metabolism and lipidomics (“lipid metabolism”, “lipidomics”, “cholesterol”, “APOE”, “sphingolipids”, “ceramides”, “polyunsaturated fatty acids”, “lipid peroxidation”, “ferroptosis”, and “lipid droplets”) with disease terms (“Alzheimer’s disease”, “Parkinson’s disease”, “Huntington’s disease”, and “amyotrophic lateral sclerosis”). Additional terms relating to biomarkers, analytical methods, multi-omics integration, and therapeutic interventions were used where relevant. We considered peer-reviewed original research articles, clinical studies, and relevant reviews addressing lipid-related mechanisms, lipidomic biomarkers, analytical approaches, and therapeutic strategies in these disorders. Clinical trial registries and relevant reports were also consulted to assess the development status and outcomes of lipid-related interventions. Studies were selected based on their relevance to the scope of the review, with attention to the distinction between human observational findings, experimental model evidence, and clinical outcomes. This review provides a narrative synthesis of the literature rather than a formal systematic review or meta-analysis; therefore, the search and study selection were intended to support a broad, mechanistically informed overview rather than an exhaustive accounting of all eligible publications.

## 2. Neuroinflammation and the Brain Lipidome

Neuroinflammation and lipid dysregulation are emerging as major contributors to the process of neurodegeneration. The lipidome of the brain is characterized by its molecular complexity and dynamic regulation and plays a major role in several neuronal processes such as neuronal function, neurotransmission, myelination, and synaptic plasticity [17,18]. This intricate lipid landscape includes different lipid classes, helps to maintain cellular membrane integrity, supports cell-to-cell communication, and serves as precursors for signaling molecules that modulate the function of neurons and glial cells [19]. For example, cholesterol is required for myelin and synaptic membranes, regulating membrane fluidity and the activity of receptors and ion channels, as well as phospholipids being integral membrane components and precursors for eicosanoids and other mediators involved in neuroinflammatory signaling. The disruption of these finely regulated lipid metabolic pathways can cause inflammatory responses and glial dysfunction, which can result in neuronal damage and contribute to neurodegeneration [20]. The pathological lipidosis and microglial activation in AD are closely linked, leading to synaptic dysfunction [21]. The apolipoprotein E ε4 (APOE4) allele is a major genetic risk factor for AD, profoundly affects the lipid metabolism of microglia and increases the levels of neuroinflammation and disease progression [22,23]. The aim of this review is to discuss the complex relationship between lipid metabolism and neuroinflammation, and how their intersection affects the pathogenesis of neurological diseases [20,24].

Neuro-immune cells, including microglia, require polyunsaturated fatty acids (PUFAs), particularly omega-3 fatty acids, for maintaining the structure and metabolic homeostasis of their cell membranes [25]. Recent evidence shows that PUFAs are recognized as active modulators of microglial activation and neuroinflammation [24,25]. This dysregulated PUFA metabolism has been shown to impair neuronal signal transduction, neurogenesis, and neuroinflammation, contributing to the pathophysiology of AD and PD [26]. Therefore, it is crucial to understand the dynamic lipidomic changes in microglia, particularly in response to inflammatory stimuli, to identify novel therapeutic targets for neurodegenerative diseases [23].

### 2.1. Major Lipid Classes in the Brain

Lipid classes are not simply required for the structure of the cellular membrane, but also play an important role in neuronal signaling, synaptic plasticity, and pathways of cellular stress and survival. For example, sphingolipids, particularly ceramides, have been suggested to play a role in stress response, inflammation, and apoptosis. In contrast, glycerophospholipids, which include phosphatidylcholine and phosphatidylethanolamine, are associated with membrane fluidity and stored lipid sources for intercellular communication [27]. Furthermore, the complex relationship between these lipid classes has a significant role in the regulation of cellular homeostasis, and imbalances in these classes may have dramatic effects on brain health.

#### 2.1.1. Cholesterol and Sterols

Cholesterol is the predominant sterol in the brain, which is essential for maintaining the membrane integrity, myelin sheaths stability and synaptic function especially in neurons and oligodendrocytes [28,29,30]. In addition, cholesterol plays an important role in the assembly of lipid rafts that are essential for clustering of receptors and signal transduction processes, which are crucial for neurotransmission and neuronal survival [31]. Apart from its structural and signaling functions, cholesterol metabolism is closely linked to neurodegenerative pathologies. Changes in cholesterol transport, synthesis, esterification and degradation may affect membrane properties, synapse formation, and influence processes associated with amyloid precursor protein processing [32]. For instance, in AD, disturbances in cholesterol homeostasis have been linked to changes in amyloid- and tau- related pathology, and genetic factors linked to changes in lipid transport [21,22]. However, the relationship between cholesterol dysregulation and neurodegeneration is complex and may differ among cell types, disease stage, and specific cholesterol metabolic pathways.

#### 2.1.2. Glycerophospholipids

Glycerophospholipids are the primary structural component of cell membranes and are responsible for the organization and functional compartmentalization of the membranes. Other important subclasses are phosphatidylcholine (PC), phosphatidylserine (PS) and phosphatidylethanolamine (PE), which also play roles in membrane fluidity and in the production of lipid mediators and modulators of intercellular communication [27]. Furthermore, glycerophospholipids are also used as a reservoir for fatty acids that can be released by phospholipases with the specific hydrolysis of the sn-2 position of the glycerol backbone, such as phospholipases A2, that are involved in several cerebral processes [11,26,33,34]. Changes in glycerophospholipid species have been previously reported in neurodegenerative conditions, although the direction and biological significance of these alterations may vary between diseases and experimental or clinical contexts.

#### 2.1.3. Sphingolipids and Ceramides

Sphingolipids are significant components of brain lipids and are particularly rich in neuronal membranes and myelin sheaths, which are essential for cell membrane stability and signaling pathways. Major sphingolipid classes in the CNS include ceramides, sphingomyelins, glycosphingolipids and gangliosides. Among these, ceramides are key bioactive molecules that regulate various cellular responses that are essential to neuronal survival, membrane remodeling, oxidative stress, inflammation and apoptosis [27].

#### 2.1.4. Polyunsaturated Fatty Acids

The CNS is enriched with polyunsaturated fatty acids, such as arachidonic acid (AA) and docosahexaenoic acid (DHA), which are involved in maintaining membrane fluidity, regulating inflammatory responses, and axonal growth [35,36,37,38]. Both DHA (omega-3 PUFA) and AA (omega-6 PUFA) can be released from membrane phospholipids through the action of phospholipases and subsequently serve as precursors for bioactive lipid mediators [39,40,41]. AA-derived metabolites include eicosanoids that participate in inflammatory signaling, whereas DHA can give rise to specialized pro-resolving mediators involved in the regulation of inflammatory responses [32,34]. Dysregulation of PUFA metabolism has been associated with neuroinflammation, oxidative stress, membrane dysfunction, and neuronal vulnerability in neurodegenerative diseases [32].

#### 2.1.5. Lipid Mediators and Lysophospholipids

The enzymatic activity of phospholipase A2 generates a range of lipid products, including lysophospholipids and free fatty acids, that are precursors of various signaling molecules such as eicosanoids and docosanoids, which regulate synaptic functions and neuroinflammatory responses [42,43]. These lipid mediators have a crucial role in regulating synaptic transmission and plasticity, which are responsible for modulation of neuronal excitability and communication [44,45]. Moreover, some of these lipid species (such as lysophospholipids) have been demonstrated to have neuroprotective properties in cerebral ischemia, which is mediated by enhancing cell membrane integrity and preventing neuronal death [46]. Conversely, perturbations in phospholipid metabolism, as a result of Aβ accumulation, can lead to the generation of lysophosphatidylcholine and the production of lipid-peroxidation products such as 4-hydroxynonenal, which can cause neurotoxicity and neurodegeneration [47].

### 2.2. Lipid Metabolism and Homeostasis in the Central Nervous System

The CNS is responsible for the regulation of lipid metabolism and lipid homeostasis. The complexity of lipid metabolism in the CNS elucidates its pivotal role in neurological function and disease [32,48]. Lipid dysregulation may result in the accumulation of toxic lipid species, resulting in neuroinflammation, demyelination, and neuronal loss in several neurodegenerative diseases [49]. However, detrimental alterations in lipid metabolism that lead to accumulation of harmful lipid species are known factors in impaired neurological outcomes and complications after CNS injuries [50]. The metabolic status of the brain can be reflected in the cerebrospinal fluid (CSF), which provides a diagnostic window for changes in lipids associated with neurological disorders [1].

The lipidome of the brain is highly complex and significantly varies across different regions, corresponding to the structural and functional properties of the brain regions [8]. Therefore, this regional lipidomic profile is important for understanding the abnormal metabolism of the affected brain regions in specific neurological diseases [51]. Moreover, the influx and efflux of lipid molecules in the brain are highly regulated by specialized lipid transport mechanisms and the blood–brain barrier (BBB), which reflect the metabolic autonomy of the brain [51]. Recent studies have focused on dysregulated lipid metabolism, which is known to play an important role in the progression of neurodegenerative disorders. In PD, imbalanced lipid homeostasis causes protein aggregation and oxidative stress [52]. In AD, impaired lipid metabolism results in the aggregation and propagation of protein aggregates and triggers neurodegeneration [53,54,55].

## 3. Lipid Dysregulation in Neurodegenerative Diseases

Lipid homeostasis disruption plays a role in the onset and progression of major neurodegenerative diseases like AD, PD, HD, and ALS [4]. Although they have distinct pathological hallmarks, accumulating evidence suggests that they share common underlying mechanisms, which involve aberrant lipid signaling, impaired lipid transport, and altered membrane dynamics [19]. In AD, there is an abnormal deposition of β-amyloid plaques and neurofibrillary tangles associated with disturbances in lipid metabolism [56]. Specifically, changes in cholesterol homeostasis have a significant role in the pathogenesis of AD, particularly in the processing of amyloid precursor protein (APP) and Aβ peptides [57]. Additionally, lipid droplets have been found to be deposited in glial cells during neurodegenerative diseases (AD and PD), which may affect protein aggregation and disease development [58,59,60]. Impaired lipid metabolism interacts with glucose dysregulation to further contribute to Aβ aggregation and tau pathology, and altered cholesterol homeostasis affects APP processing, and ApoE4 exacerbates deficits in Aβ clearance [61,62]. Likewise, PD is now considered a proteinopathy, and α-synuclein toxicity and defects in cell trafficking are closely related to changes in lipid droplet accumulation, polyunsaturated fatty acids peroxidation, and disrupted sphingolipid metabolism, all of which contribute to α-synuclein aggregation, mitochondrial dysfunction, and lysosomal impairment [63,64]. Similarly, HD results in the abnormal huntingtin protein, which disrupts sterol regulatory element-binding proteins (SREBPs), which in turn leads to impaired cholesterol biosynthesis and a change in phospholipid composition, ultimately causing an increase in neuronal dysfunction [61]. In addition, ALS is associated with systemic lipid alterations, including changes in fatty-acid profiles and sphingolipid metabolism that contribute to neuroinflammation and motor-neuron degeneration [59]. These metabolic changes highlight the diverse and complex functions of lipids in neurodegenerative diseases [58,59,65]. For conceptual clarity, this review groups lipid-related findings into four interconnected areas: cholesterol and APOE, sphingolipids, PUFAs, and lipid droplets. These areas are not intended to represent a single established causal pathway common to all neurodegenerative diseases. Instead, they provide a framework for comparing evidence concerning lipid transport, membrane organization, lipid signaling, oxidative injury, and cellular lipid storage. The relative contribution of each process may differ by disease, brain region, cell type, disease stage, and lipid species. Human observational findings are therefore interpreted as associations unless supported by evidence establishing a causal relationship, while mechanistic conclusions derived from cellular and animal models are identified as experimental evidence requiring appropriate clinical validation. The progression of neurodegenerative pathology is driven by the convergence of four interconnected lipid metabolic pathways: cholesterol–APOE signaling, sphingolipid metabolism, PUFA dynamics, and lipid droplet formation.

**Figure 1:**
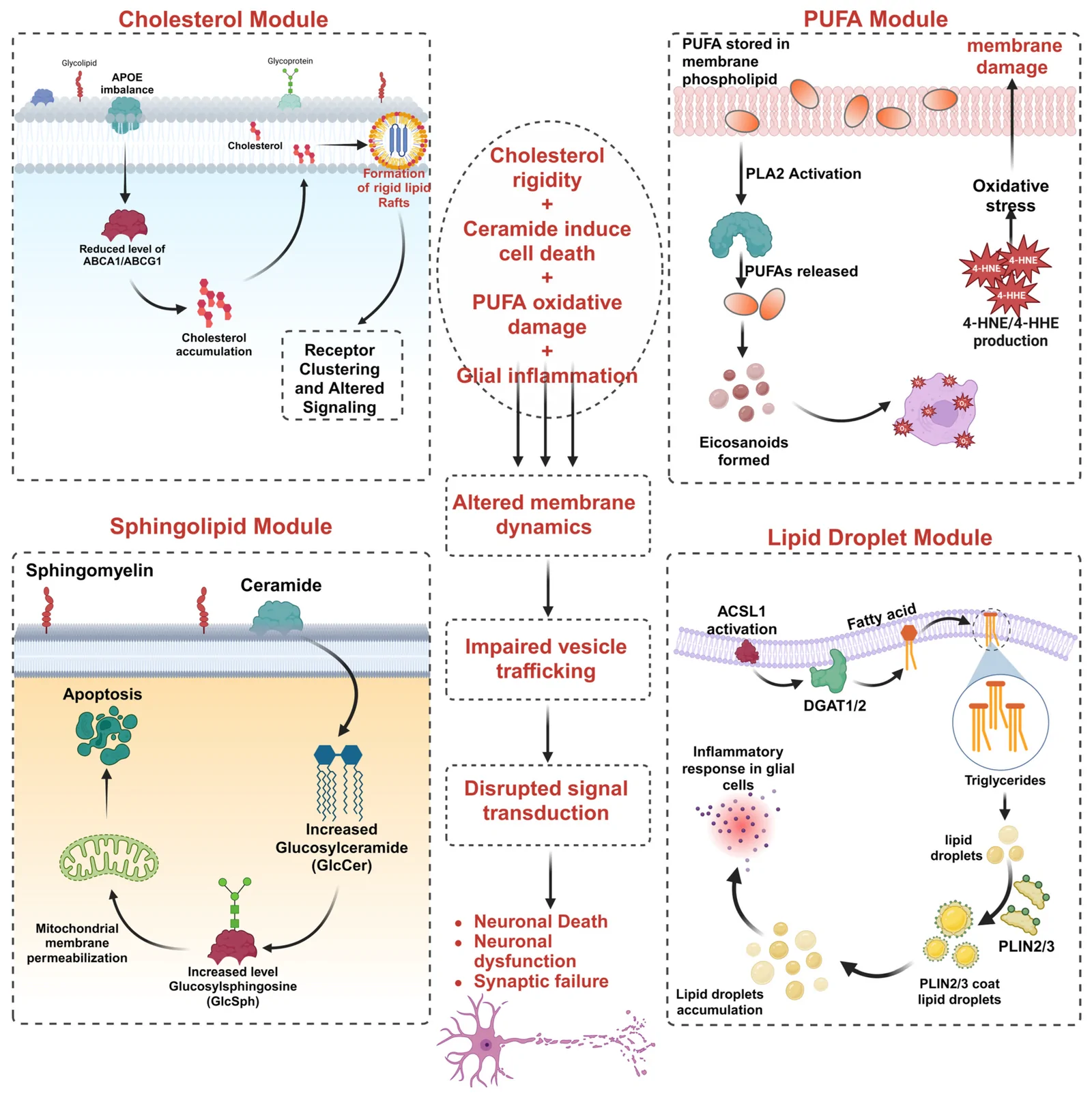
Lipid metabolism cascades in neurodegenerative pathology. Schematic presentation of neuronal and glial dysfunction is converged by four different lipid metabolic pathways, including cholesterol, sphingolipid, polyunsaturated fatty-acid (PUFA) and lipid droplet dynamics. Cholesterol–APOE module: efflux of cholesterol mediated by ABCA1/ABCG1 is impaired by the APOE4 isoform, leading to cholesterol accumulation, rigid lipid-raft formation, and aberrant receptor clustering that disrupts transmembrane signaling. Sphingolipid module: disrupted sphingomyelin homeostasis increases ceramide levels and its metabolites GlcCer, GlcSph, and triggers mitochondrial outer membrane permeabilization that causes apoptosis. PUFA module: neuroinflammatory or oxidative stimuli activate phospholipase A2, releasing PUFAs from membrane phospholipids. Their overproduction of lipid peroxidation generates reactive aldehydes, including 4-HNE and 4-HHE, that increase oxidative stress and damage membrane integrity. Lipid droplet module: chronic metabolic stress activates ACSL1 and DGAT1/2, promoting fatty-acid esterification into triglycerides and their storage in PLIN2/3 coated lipid droplets. Excessive lipid droplet accumulation promotes neuroinflammation in glial cells. The interaction of membrane rigidity, lipotoxicity, and inflammatory signaling disrupts membrane dynamics and vesicle trafficking, driving synaptic dysfunction, neuronal loss, and ultimately cognitive and motor impairment.

In the cholesterol module, the APOE4 isoform is associated with impaired cholesterol homeostasis and reduced efficiency of ABCA1/ABCG1-mediated cholesterol efflux. These alterations may promote abnormal cholesterol accumulation in cellular membranes, rigid lipid raft formation, and disrupt membrane-associated signaling pathways, thereby contributing to neurodegenerative processes. At the same time, the sphingolipid module is disrupted from the ceramide-S1P rheostat; an increased flux of ceramides and metabolites such as glucosylceramide triggers mitochondrial outer membrane permeabilization, which serves as a critical step initiating apoptosis [66,67,68]. In the PUFA module, oxidative stress or neuroinflammatory stimuli activate phospholipase A2 to release fatty acids such as DHA and AA from the bilayer; these species then undergo a lipid-peroxidation chain reaction to produce reactive aldehydes like 4-hydroxy-2-nonenal (4-HNE) that compromise membrane integrity [69,70,71]. Chronic metabolic stress also includes the activation of ACSL1 and DGAT1/2, facilitating the esterification of fatty acids into triglycerides in PLIN-coated lipid droplets and promoting a pro-inflammatory phenotype that leads to systemic neuroinflammation [19,72,73,74,75]. These pathways act together to cause impaired vesicle trafficking, synaptic degradation, and neuronal loss [19,68,72,76,77] as shown in Figure 1.

### 3.1. Alzheimer’s Disease

**Figure 2:**
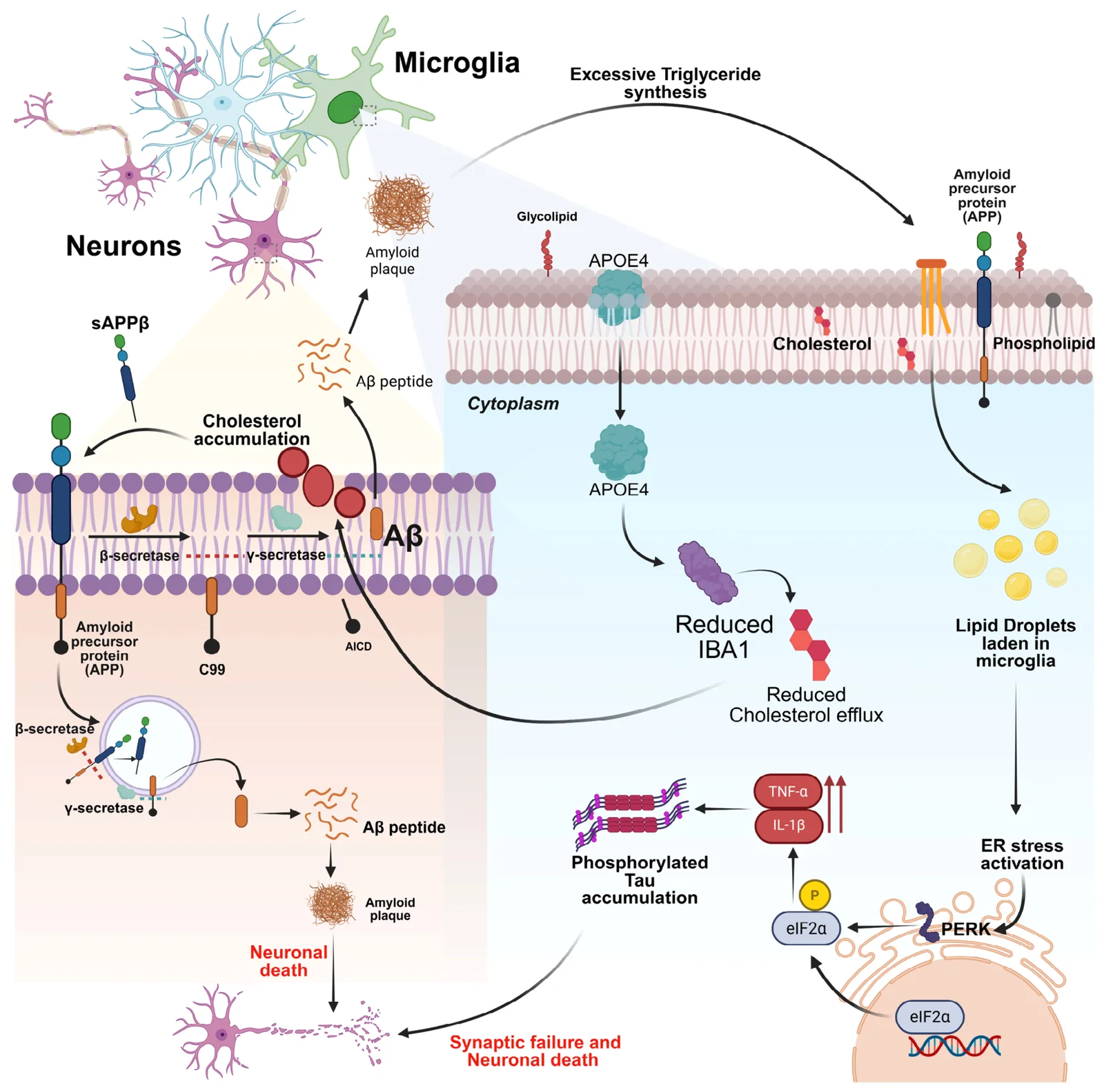
APOE4-driven dysregulation of lipid metabolism links amyloid pathology, microglial dysfunction, and neurodegeneration. Schematic representation of the proposed mechanism in which APOE4 alters cholesterol and lipid handling within neurons and microglia, leading to the pathogenesis of AD. In neurons, amyloid precursor protein (APP) is sequentially cleaved by β-secretase and γ-secretase, generating soluble APPβ (sAPPβ) and amyloid-β (Aβ) peptides that can accumulate and contribute to plaque formation, synaptic dysfunction, and neuronal death. In microglia, APOE4-dependent alterations in lipid transport may contribute to intracellular lipid accumulation and lipid droplet formation. Impaired cholesterol efflux through transporters such as ABCA1 and ABCG1 may further disturb cellular lipid homeostasis. In addition, lipid overload further causes endoplasmic reticulum (ER) stress and activation of the PERK–eIF2α pathway, which leads to the release of inflammatory cytokines (TNF-α, IL-1β) and phosphorylated tau accumulation. Collectively, defects in lipid metabolism caused by APOE4 worsen the amyloid and tau pathology, microglial inflammation, and progressive synaptic failure and neuronal loss.

Lipid dysregulation is increasingly recognized as an important component of AD pathobiology, particularly through interactions among cholesterol transport, APOE genotype, membrane organization, APP processing, Aβ clearance, and neuroinflammation [78,79]. APOE4-associated alterations in cholesterol transport and phospholipid handling in astrocytes and microglia can modify membrane lipid organization and pathological lipid-raft properties, potentially influencing APP processing and amyloid pathology [80,81]. These shifts in biophysical properties of rafts may influence APP processing by altering membrane organization and the localization or activity of proteins involved in amyloidogenic and non-amyloidogenic APP cleavage, potentially affecting Aβ generation [81,82]. Microglia upregulate the enzyme ACSL1 and increase triglyceride production, leading to the formation of lipid droplet-laden microglia-active organelles that exhibit a chronic pro-inflammatory state and compromised phagocytic potential [78,79]. Chronic cellular stress activates the PERK-eIF2α axis, further stimulating the release of pro-inflammatory cytokines such as TNF-α and IL-1β and promoting Tau hyperphosphorylation, synaptic failure and neuronal loss [83,84,85,86], as shown in Figure 2.

### 3.2. Parkinson’s Disease

PD is associated with alterations in sphingolipid metabolism and lysosomal function. Pathogenic variants in GBA1 or reduced glucocerebrosidase (GCase) activity can alter the metabolism and lysosomal handling of glycosphingolipids, including glucosylceramide and glucosylsphingosine. Experimental studies suggest that these lipid alterations may influence α-synuclein (α-syn) aggregation, membrane interactions, and lysosomal processing; however, the magnitude and direction of these effects may depend on the lipid species, experimental model, and cellular context [87,88,89]. Although cellular and animal studies support interactions between glycosphingolipid metabolism and α-syn pathology, the extent to which these interactions directly contribute to disease progression in humans remains incompletely established. Conversely, α-syn accumulation may further disrupt lysosomal function and intracellular trafficking, including endoplasmic reticulum-to-Golgi transport of GCase, potentially reducing its delivery to lysosomes and exacerbating lipid metabolic disturbances [10,89]. Furthermore, α-syn interacts with synaptic vesicle-associated proteins, including vesicle-associated membrane protein 2 (VAMP-2), and may alter soluble N-ethylmaleimide-sensitive factor attachment protein receptor (SNARE)-dependent vesicle trafficking and neurotransmitter release [90,91,92].

Lipid–protein interactions are governed by specific structural regions of proteins and by the physicochemical properties of the lipid membrane. In PD, α-syn provides a well-characterized example: its N-terminal region (approximately residues 1–95), which contains repeated KTKEGV motifs, mediates membrane association, and adopts an amphipathic α-helical conformation upon binding. Positively charged residues, including lysines, contribute to interactions with negatively charged phospholipid headgroups, while membrane curvature and lipid acyl-chain composition influence binding affinity and protein conformation [93]. The central non-amyloid-β component (NAC; approximately residues 61–95) overlaps with this membrane-interacting region and contributes to aggregation, linking membrane association to α-syn assembly under experimental conditions. These interactions are not determined by protein sequence alone: lipid composition, membrane curvature, and the relative abundance of free and membrane-bound protein can alter the balance between physiological membrane association and pathological aggregation. Collectively, these findings suggest a potentially bidirectional relationship between lipid dysregulation, lysosomal dysfunction, and α-syn pathology, although the causal contribution of these interactions to human PD progression requires further investigation.

**Figure 3:**
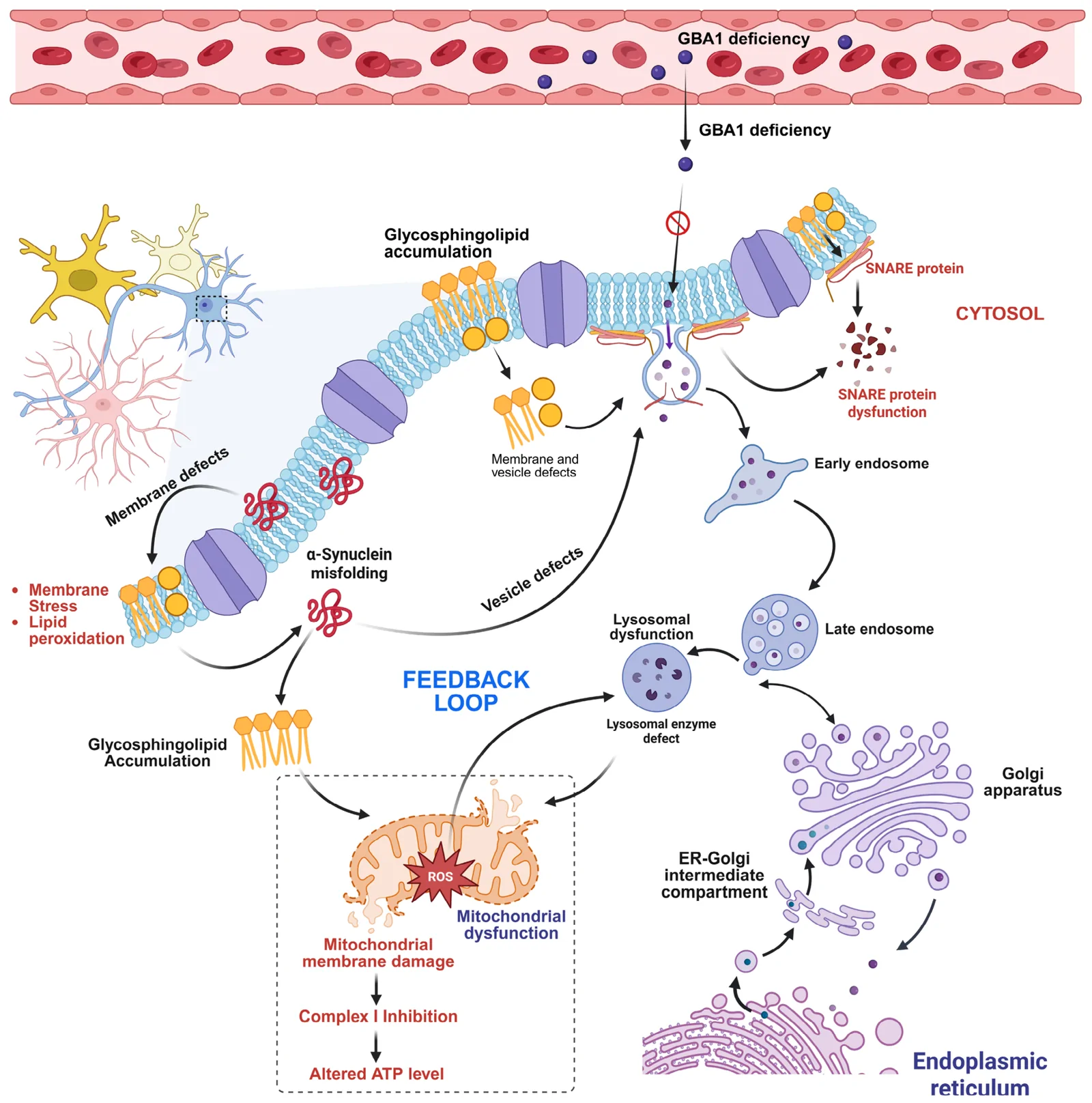
Proposed interactions between GBA1 deficiency, lysosomal dysfunction, and α-synuclein pathology. GBA1 deficiency or reduced glucocerebrosidase activity may alter lysosomal glycosphingolipid metabolism and influence α-synuclein handling. Experimental evidence suggests that changes in lipid composition can interact with membrane properties, vesicular trafficking, lysosomal activity, and mitochondrial function. These processes may reinforce one another and contribute to cellular stress. However, the strength and direction of individual relationships vary across experimental models, and not all proposed links have been established causally in human Parkinson’s disease.

Chronic membrane stress triggers the peroxidation of PUFA species like DHA and AA, generating reactive aldehydes such as 4-HNE, which stabilize α-syn oligomers and induce mitochondrial dysfunction by inhibiting Complex I, resulting in ATP depletion and oxidative stress [94,95,96]. The resulting vicious cycle of lipid dyshomeostasis and proteinopathy ultimately leads to the selective degeneration of dopaminergic neurons and the development of characteristic motor symptoms [97], as shown in Figure 3.

### 3.3. Huntington’s Disease

Alterations in lipid metabolism, including changes in PUFA-containing phospholipids, have been reported in HD and may contribute to altered membrane integrity, synaptic function, lipid peroxidation, and neuroinflammatory signaling. Lipid dysregulation in HD includes reported changes in phospholipid composition, fatty-acid metabolism, and cholesterol homeostasis [98,99,100,101]. These alterations may affect membrane organization, synaptic function, and neuronal vulnerability, although their direction and magnitude can differ among brain regions, disease stages, and experimental systems. PUFA-containing membrane phospholipids are particularly relevant to oxidative injury because their unsaturated fatty-acid chains are susceptible to lipid peroxidation [98]. Experimental studies in HD models have implicated iron-dependent lipid peroxidation and ferroptosis-related pathways in mutant-huntingtin-associated cellular injury. Particularly, ALOX5-mediated ferroptosis has been reported in experimental HD systems, although the contribution of this pathway varies with the experimental conditions and model [102]. However, lipid peroxidation and ferroptosis-related pathways represent candidate mechanisms for further investigation rather than established, disease-specific therapeutic targets in humans. Therapeutic studies targeting fatty-acid composition or lipid oxidation require careful interpretation. For example, deuterium-reinforced linoleic acid has been investigated as a strategy to reduce lipid peroxidation in experimental models, including the Q140 knock-in mouse model of HD. In this model, dietary deuterium-reinforced linoleic acid reduced striatal lipid-peroxidation products and improved performance in a novel-object-recognition task but did not significantly improve motor deficits or reduce huntingtin aggregation [98]. These findings provide a mechanistic rationale for investigating the role of oxidizable PUFAs in HD; however, efficacy in an animal model does not establish clinical benefits in patients. Likewise, omega-3 PUFA supplementation has been discussed as a potential approach for alleviating HD symptoms, but it should not be described as an established disease-modifying treatment without adequate supporting clinical evidence [103]. However, the extent and direction of specific lipid alterations may vary across brain regions, disease stages, and experimental or clinical populations [98,99,100]. Therapeutically, omega-3 polyunsaturated fatty acids (n-3 PUFAs) have been investigated as potential therapeutic approaches in reducing the severity of HD, although available evidence remains insufficient to establish their clinical efficacy [103]. Clinical and epidemiological studies have investigated the potential neuroprotective effect of the omega-3 fatty acids in neurodegenerative diseases. However, evidence for clinical benefit remains variable across diseases and study populations [104,105].

### 3.4. Amyotrophic Lateral Sclerosis

In ALS, altered lipid metabolism has been associated with changes in circulating lipid profiles, fatty-acid handling, membrane composition, and oxidative stress [106,107,108]. Sphingolipids, including ceramides and gangliosides, contribute to neuronal membrane organization and cellular signaling, and dysregulated sphingolipid metabolism has been implicated in ALS-related processes [109]. However, the extent to which individual lipid alterations contribute directly to motor-neuron degeneration, rather than arising secondarily during disease progression, remains uncertain [107,110]. PUFA-containing phospholipids are susceptible to lipid peroxidation, which may compromise membrane integrity and contribute to cellular dysfunction. Ferroptosis-related mechanisms have attracted interest in neurodegeneration, but evidence from other neurological disorders should not be treated as direct proof of an equivalent causal mechanism in human ALS. ALS-specific evidence and the translational limitations of current findings require careful consideration [107,108].

The therapeutic implications of lipid-peroxidation pathways remain under investigation. RT001, a deuterated synthetic analog of linoleic acid designed to make membrane PUFAs more resistant to lipid peroxidation, was evaluated in a randomized, placebo-controlled phase 2 study involving 43 participants with ALS. At 24 weeks, the between-group difference in the ALS Functional Rating Scale-Revised (ALSFRS-R) score was not statistically significant (least-squares mean difference 1.90; 95% confidence interval −1.39 to 5.19; *p* = 0.25). Although RT001 was generally well tolerated, these findings do not establish clinical efficacy and indicate the need for a larger clinical trial [111]. Further research is needed to determine whether specific lipidomic signatures or lipid-peroxidation pathways can serve as validated biomarkers or therapeutically actionable targets in ALS.

### 3.5. Comparative Synthesis Across Diseases

Across AD, PD, HD, and ALS, lipid dysregulation involves overlapping biological processes, but the consistency of the evidence varies by lipid class and disease. Alterations in cholesterol handling and APOE-related pathways are particularly prominent in AD, whereas sphingolipid metabolism and GCase dysfunction have a well-established genetic and mechanistic connection to subsets of PD. In HD, changes in phospholipid composition, fatty-acid metabolism, and cholesterol homeostasis have been reported, alongside experimental evidence implicating lipid peroxidation and ferroptosis-related pathways. In ALS, altered circulating lipid profiles, fatty-acid handling, and sphingolipid metabolism have been described, although the contribution of individual lipid species to motor-neuron degeneration remains uncertain [87,88,89,98,99,100,102,106,107,108,109,110,112,113]. These findings should not be interpreted as evidence that the same lipid changes occur in all four diseases. The direction and magnitude of reported changes may vary with disease stage, brain region, biological matrix, participant characteristics, and analytical method. Accordingly, some lipid pathways are supported by convergent evidence, whereas other proposed relationships remain inconsistent or predominantly experimental. Comparative interpretation requires disease-specific validation and careful distinction between human associations and mechanistic findings from cellular or animal models.

## 4. Lipidomics for Biomarker Discovery

Lipidomics, the comprehensive analysis of lipids and their metabolic pathways, provides a valuable tool for identifying novel lipid-based biomarkers that can help in the early diagnosis, prognosis, and monitoring of therapeutic response in neurodegenerative diseases [9]. Lipids form a very large and diverse class of molecules, containing thousands of different lipid molecules that could serve as potential markers for early biochemical changes before the onset of clinical symptoms [106]. Unlike genomics or proteomics, lipidomics provides a dynamic picture of cellular physiology that reflects both genetic predisposition and environmental influences [3]. These dynamic characteristics of lipid biomarkers allow for the identification of minor changes that can occur at onset or progression of disease, even prior to irreversible neuronal damage. Furthermore, alterations in plasma lipid profiles, particularly glycerophospholipids, have emerged as potential diagnostic and prognostic markers in ALS [106]. In AD, several lipid markers have been associated with disease severity and progression, including ceramide and oxidized phospholipid changes. However, which could help to monitor disease trajectory, or therapeutic efficacy remains investigational and requires validation in independent longitudinal cohort [114]. Likewise, the detection of specific lipid classes in CSF, such as gangliosides and sulfatides, has been identified as a potential method for early diagnosis of PD, prior to the onset of a motor phenotype [1]. Although several lipid species and lipid signatures have emerged as candidate biomarkers, many remain at the research or validation stage and require replication in larger, independent, and clinically diverse cohorts to establish their diagnostic or prognostic utility before routine clinical application [115,116].

### 4.1. Targeted Lipidomic Approaches

Targeted lipidomic approaches are essential in many neurodegenerative diseases, such as immune disorders, redox imbalance, autophagy dysfunction, and dysregulation in iron homeostasis [108]. The lipid mediators involved in inflammation and mitochondrial function may have potential use for quantification and may be relevant to metabolic reprogramming associated with immunosenescence and neuroinflammation in neurodegeneration [112]. These targeted analyses allow for the identification of specific lipid signatures which may lead to diagnosis and prediction of disease progression [117,118]. Additionally, targeted mass spectrometry of CSF samples, blood, and exosomes has resulted in the identification of novel lipid signatures for ALS, which could be used to aid development of better diagnostic and prognostic tools [119,120]. Beyond diagnosis, these fluid-based biomarkers can be employed to detect disease activity and response to therapy in various neurodegenerative diseases, including AD, PD, and multiple sclerosis. [56,121]. These advances in lipidomics offer promising avenues for personalized medicine, allowing for the potential stratification of patients by their distinct lipid profiles and the development of more effective and personalized therapeutic strategies [10,121]. In addition to identification, these lipid changes need to be functionally characterized to enable the development of therapies that could modulate dysregulated lipid pathways [122]. Targeted lipidomics is a quantitative approach that measures predefined lipid species using validated analytical methods, commonly liquid chromatography–tandem mass spectrometry (LC–MS/MS). Multiple reaction monitoring (MRM) selectively detects and quantifies specific lipid molecules by monitoring characteristic precursor-to-product ion transitions. Stable-isotope-labeled or structurally similar internal standards are added to correct for analytical variability, extraction losses, and matrix effects. When calibrated with appropriate reference standards, targeted lipidomics enables absolute quantification, expressed as lipid concentration per unit volume or mass, rather than relative abundance alone.

### 4.2. Untargeted Lipidomic Approaches

The combination of advanced mass spectrometry technologies and untargeted lipidomics provides a broad view of lipidome changes in pathophysiological conditions [123]. This unbiased approach is especially useful for neurodegenerative diseases where the lipid dysregulations are pleiotropic and often involve many interconnected pathways. This can be useful for identification of new lipid classes or unanticipated metabolic changes that might not be identified by targeted methods, thereby giving insight into disease mechanisms [120]. The comprehensive lipid profiles obtained by untargeted lipidomics can be especially helpful in characterizing complex lipid signatures that allow for the differentiation of various neurodegenerative diseases like AD and PD, which could help in differential diagnosis. Moreover, combining untargeted lipidomics with other omics studies, such as proteomics and metabolomics, can provide more comprehensive pictures of lipid metabolism, revealing the complex interactions with other cellular processes and providing a deeper understanding of disease pathogenesis [115]. Such a comprehensive approach can reveal novel therapeutic targets by identifying disrupted lipid pathways that play a role in neuroinflammation, oxidative stress, and mitochondrial dysfunction in neurodegeneration [4]. Untargeted lipidomics analysis can also identify new potential disease progression and therapeutic response monitoring biomarkers, offering a robust platform for drug discovery and personalized medicine [124].

Importantly, statistical differences in lipid abundance alone are insufficient to establish a clinically useful biomarker. Diagnostic studies should evaluate sensitivity, specificity, receiver operating characteristic (ROC) curves, area under the curve (AUC), positive predictive value (PPV), and negative predictive value (NPV) in clinically relevant cohorts that include appropriate neurological controls and different disease stages [125,126,127]. Multi-lipid signatures may provide greater diagnostic information than individual lipid species because neurodegenerative diseases involve multiple interconnected metabolic pathways [9,128]. Untargeted lipidomics is particularly valuable for biomarker discovery, whereas targeted lipidomics can provide sensitive and quantitative validation of selected candidates [127]. Integration of lipidomic signatures with clinical, imaging, genetic, or proteomic biomarkers may further improve disease classification and patient stratification [9]. Differences between targeted and untargeted lipidomics in terms of analytical approach, quantification, applications, advantages, limitations, and validation requirements are summarized in Table 1.

## 5. Therapeutic Strategies Targeting Lipid and Membrane Associated Mechanisms

The therapeutic intervention targeting lipid metabolism represents a multifaceted approach to treating neurodegenerative disease by addressing underlying molecular pathology [129]. This includes strategies to restore lipid homeostasis, reduce lipid-mediated inflammation, and activate lipid-dependent neuroprotective mechanisms [116,130]. Therefore, pharmacological modulation of key enzymes that regulate sphingolipid synthesis (e.g., ceramidases and sphingomyelin synthases) can restore ceramide and sphingosine-1-phosphate to normal levels, which may have a regulatory effect on neuronal function and survival [131,132]. However, the approach that targets lipid transport and clearance mechanisms, particularly apolipoproteins and lipid-binding proteins, could also provide a potential pathway to mitigate pathological lipid accumulation and facilitate effective waste removal in the brain [14]. Recent evidence suggested the importance of pro-resolving mediators in actively resolving neuroinflammation and supporting tissue repair by regulating lipid pathways [133]. In this context, APOE-directed strategies have been investigated to enhance Aβ clearance. Bexarotene, a retinoid X receptor agonist, initially showed promising effects in preclinical AD models; however, subsequent studies did not consistently reproduce its effects on amyloid pathology, and a small clinical study did not demonstrate an overall reduction in brain amyloid burden [134,135,136,137]. Compounds that elevate brain ApoE levels, such as bexarotene and CS6253, are designed to improve the clearance of Aβ, highlighting the therapeutic potential of lipid-based interventions [56]. Alterations in ceramide metabolism have been associated with changes in APP processing, Aβ accumulation, and tau pathology. Therefore, modulation of ceramide-associated pathways may represent a potential therapeutic strategy, although the precise mechanisms and therapeutic direction require further validation [113,138,139,140]. Lipid dysregulation has a wide range of consequences, including Aβ deposition, clearance and association with metabolic disorders like diabetes and hypercholesterolemia; therapeutic strategies should take the broader metabolic context [27,141]. Overall, these approaches remain under investigation, and their therapeutic potential requires further validation in disease-specific preclinical models and well-designed clinical studies.

### 5.1. Modulation of Lipid Synthesis and Degradation

Targeting specific enzymes involved in sphingolipid metabolism is a therapeutic approach. Inhibiting ceramide synthases or increasing ceramidase activity may reduce pro-apoptotic ceramide levels, thereby decreasing neuronal damage [133]. In parallel, strategies that target diacylglycerol kinase modulators or enzymes involved in cholesterol biosynthesis (such as HMG-CoA reductase) and sphingosine kinase 1 promote neuroprotective lipid species. Therefore, inhibitors of sphingomyelin phosphodiesterases (SMPDEs) have been suggested as therapeutic agents, because elevated sphingomyelinase (SMase) activity is associated with increased production of the amyloidogenic form of APP [142,143]. In addition, dietary omega-3-rich interventions also have the potential to prevent synaptic dysfunction in AD by inhibiting sphingomyelin metabolism and production of ceramide, which protects neurons [144].

### 5.2. Dietary and Nutritional Interventions

Nutritional approaches focused on the inclusion of omega-3 (ω-3) and omega-6 (ω-6) PUFAs may influence synaptic function, neurogenesis, membrane organization, and inflammatory signaling, although their effects depend on the specific fatty-acid species and biological context [145,146]. DHA, an ω-3 PUFA and AA, an ω-6 PUFA, contribute to neuronal membrane structure and serve as precursors for bioactive lipid mediators. Their effects on oxidative stress and neuroinflammation may vary according to their metabolism, availability, and the balance of downstream lipid mediators [146]. Specific lipid formulations, including lysophosphatidylcholine (LPC)-based delivery systems that facilitate DHA transport across the BBB through the MFSD2A transporter, have been investigated as potential approaches to improve DHA availability; however, their therapeutic benefits in AD require further clinical validation [147]. Alterations in membrane lipid composition, including changes in long-chain ω-3 PUFAs such as DHA, have been associated with synaptic dysfunction and changes in membrane properties that may influence protein trafficking and neuronal receptor function [148,149]. Altered lipid-raft composition may also affect the localization and activity of proteins involved in APP processing, potentially influencing Aβ production [148]. Although maintaining an appropriate balance of essential fatty acids is important for normal physiological function, the ω-6:ω-3 fatty-acid ratio should not be considered a validated standalone therapeutic target for neurodegenerative diseases without supporting clinical evidence [150]. Therefore, nutritional and pharmacological interventions should be evaluated using disease-relevant outcomes and controlled clinical studies rather than inferring therapeutic benefit solely from changes in fatty-acid abundance.

### 5.3. Pharmacological Approaches to Restore Lipid Homeostasis

Pharmacological interventions are designed to specifically target key enzymes and receptors involved in lipid metabolism. For example, statins inhibit 3-hydroxy-3-methylglutaryl coenzyme A (HMG-CoA) reductase, the rate-limiting enzyme in endogenous cholesterol biosynthesis. Although statins are widely used to lower circulating cholesterol, their effects on the CNS cannot be inferred directly from their peripheral lipid-lowering effects. Brain cholesterol metabolism is substantially compartmentalized from peripheral cholesterol metabolism, and the extent of CNS exposure varies among statins according to their physicochemical properties and ability to cross the BBB [57,151]. Consequently, the potential effects of statins on neurodegenerative disease mechanisms may depend on the specific compound, treatment timing, disease context, and additional biological effects. Although observational studies have reported associations between statin use and reduced dementia risk, randomized controlled trials have not established a consistent benefit in preventing dementia or slowing cognitive decline. Therefore, current evidence is insufficient to support a uniform disease-modifying effect of statins in neurodegenerative diseases [152,153]. Novel therapeutic targets include modulators of ω-3 PUFA-derived specialized pro-resolving mediators, including hydroxy-DHA, which exhibit potent anti-inflammatory and neuroprotective properties [154,155,156]. In addition, recent studies explore the therapeutic potential of reactivating protein phosphatase 2A (PP2A), an important enzyme found to be inhibited in neurodegenerative disorders, for the restoration of appropriate tau phosphorylation and the reduction in neurotoxicity [157]. Moreover, taxane derivatives such as TPI-287, a blood–brain barrier-penetrant microtubule stabilizer, were investigated as potential treatments for tauopathies; however, clinical trials reported severe hypersensitivity reactions in patients with Alzheimer’s disease and clinical worsening in patients with PSP or CBS, limiting support for further development in these populations [56,158].

Beyond these approaches, the inhibition of Stearoyl-CoA desaturase has been shown to be beneficial in animal models of AD, leading to improved cognitive function and decreased immune and synaptic impairments through modulation of key pathways involved in the pathogenesis of AD in the hippocampus [159]. The overall objective of this strategy is to counteract the damaging accumulation of reactive aldehydes associated with neuropathology in neurodegenerative diseases, which affect neuronal function [13]. These interventions, whether dietary or pharmacological, are essential for restoring lipid homeostasis, which is an important element in preventing and ameliorating the progression of neurodegenerative pathologies [24,25]. These pharmacological developments span multiple experimental stages, including in vitro mechanistic studies, in vivo validation models, and clinical-stage investigations.

#### 5.3.1. In Vitro Strategies

In vitro, studies have demonstrated that membrane-active agents, such as aminosterols (e.g., Trodusquemine and Squalamine), can remove toxic oligomers from lipid bilayers and inhibit the cytotoxicity caused by membrane disruption [160,161]. Table 2. In addition, there are also oligomer modulators (such as Anle138b), which can prevent protein-induced poration of anionic lipid membranes. Likewise, GLP-1 receptor agonists such as semaglutide restore BBB lipid integrity by reinforcing astrocyte–vascular junctional interactions. These mechanisms are further extended by emerging exosome-targeted approaches that affect disease-specific lipid and phosphoproteomic signatures within extracellular vesicles. Collectively, these findings demonstrated the central role of lipid-membrane preservation and lipid–protein interaction as common therapeutic strategies across various therapeutic classes. Therapeutic candidates were considered for inclusion according to the experimental setting and the relevance of their mechanism to lipid biology. The core tables prioritize interventions for which published evidence describes a direct effect on lipid synthesis, degradation, transport, storage, membrane composition, lipid peroxidation, or lipid-associated protein interactions. Table 2 summarizes in vitro evidence.

#### 5.3.2. In Vivo Strategies

Compounds such as Minzasolmin and Anle138bhave been shown to prevent α-synuclein-mediated lipid-membrane disruption in an in vivo model [95,170]. Table 3. GLP-1 receptor agonists such as semaglutide and liraglutide protect BBB and cerebral vascular lipid architecture, while antioxidants (α-lipoic acid) inhibit lipid peroxidation and restore mitochondrial redox balance in neurodegenerative disease models. Moreover, urolithin A stabilizes mitochondrial membrane lipid integrity through SIRT1/mTOR-mediated mitophagy and autophagic regulation, and exosome-targeted lipid-vesicle signaling pathways. Collectively, these in vivo studies demonstrated the stabilization of membrane lipid integrity and the regulation of lipid–protein interactions as central therapeutic mechanisms for neuroprotection across diverse pharmacological classes. Therapeutic candidates were considered for inclusion according to the experimental setting and the relevance of their mechanism to lipid biology. The core tables prioritize interventions for which published evidence describes a direct effect on lipid synthesis, degradation, transport, storage, membrane composition, lipid peroxidation, or lipid-associated protein interactions. In vivo evidence is summarized in Table 3.

#### 5.3.3. Clinically Evaluated Lipid-Targeted Therapeutics in Neurodegeneration

Clinical trial outcomes highlight the importance of distinguishing mechanistic plausibility from demonstrated clinical benefit. In AD, Phase 3 EVOKE and EVOKE+ trials of oral semaglutide did not demonstrate statistically significant reduction in clinical disease progression over 104 weeks [175]. In PD, Phase 2a ORCHESTRA study of minzasolmin, an investigational inhibitor of α-syn misfolding, did not meet its primary or secondary clinical endpoints; the extension program was subsequently terminated [176]. These findings illustrate that biological rationale or changes in disease-associated biomarkers do not necessarily translate into clinical efficacy.

Several clinical studies have investigated interventions that modify glycosphingolipid metabolism, PUFA biology, cholesterol regulation, or lipid peroxidation. Their outcomes illustrate the distinction between mechanistic rationale, target engagement, and demonstrated clinical efficacy. In GBA1-associated PD, venglustat, a glucosylceramide synthase inhibitor designed to reduce glycosphingolipid synthesis, was evaluated in the Phase 2 MOVES-PD trial. The intervention did not demonstrate a significant clinical benefit over placebo [177]. Ambroxol, a pharmacological chaperone of glucocerebrosidase (GCase), was investigated in a randomized clinical trial involving patients with Parkinson disease dementia. Although target engagement was demonstrated, a benefit on the primary cognitive outcomes was not established [178]. These findings support continued investigation of the GCase pathway while indicating that modification of glycosphingolipid metabolism does not necessarily translate into clinical improvement. In AD, a randomized trial of DHA supplementation did not demonstrate a significant overall slowing of cognitive or functional decline [179]. Similarly, the TREND-HD randomized trial found no significant benefit of ethyl-eicosapentaenoic acid (ethyl-EPA) over placebo on the primary motor outcome at six months [180]. These findings caution against assuming that the biological importance of omega-3 PUFAs or their proposed effects on membrane composition are sufficient to establish clinical efficacy [103,104,105].

Bexarotene, a retinoid X receptor agonist that can influence APOE-associated lipid transport and amyloid-β clearance, was investigated as a potential treatment for AD. Although initial preclinical findings were encouraging [134], subsequent studies did not consistently reproduce the reported effects [135,136]. A randomized, placebo-controlled proof-of-concept trial in moderate AD did not establish a clear overall therapeutic benefit [137]. These findings highlight the importance of independent replication before preclinical observations are interpreted as evidence of clinical efficacy. However, cholesterol in the adult brain is largely synthesized and regulated within the central nervous system, and the ability of individual statins to cross the BBB varies [29,31,151]. Randomized evidence has not established that statins prevent dementia or consistently improve established cognitive impairment [152,153]. Therefore, statins should not be presented as established disease-modifying treatments for AD solely based on their cholesterol-lowering effects. Deuterated PUFAs represent an alternative strategy intended to reduce lipid peroxidation. In the Q140 knock-in mouse model of HD, deuterium-reinforced linoleic acid reduced lipid peroxidation and mitigated cognitive impairment, although not all disease-related outcomes improved [98]. In contrast, RT001, a deuterated synthetic analog of linoleic acid, was evaluated in a randomized Phase 2 trial involving participants with ALS. The study did not establish statistically significant clinical efficacy [111]. Collectively, these findings indicate that lipid-related pathways are therapeutically relevant research targets, but clinical outcomes vary by intervention, disease, and outcome measure. Larger, adequately powered trials and confirmation of relevant target engagement are needed before these approaches can be considered established treatments. Human clinical investigations are summarized in Table 4.

## 6. Translational Lipidomics in Neurodegeneration: Biomarkers and Personalized Medicine

Despite significant advancements in lipidomics, there are still challenges to achieve a comprehensive understanding of these lipid alterations in neurodegeneration and translate these findings into clinical interventions. The complex and diverse lipid molecules require advanced analytical techniques with enhanced sensitivity and specificity for comprehensive lipidomic profiling [183]. Moreover, there is a need to investigate complex and low-abundance lipid species rather than well-characterized lipid classes, as some unidentified lipids may play critical roles in the pathophysiology of neurological diseases [184]. In addition, there is also another major challenge in data interpretation and integration due to the limited availability of systematic and standardized lipid databases [185]. Therefore, the standardization of lipidomic methodologies is essential to ensure data consistency, reliability, comparability, and reproducibility, which facilitates the translation of lipidomics into clinical interventions [186].

### 6.1. Biofluid-Based Lipid Biomarkers (CSF, Plasma and Exosomes)

CSF, plasma/serum, and extracellular-vesicle lipid profiles should not be interpreted as biologically equivalent. CSF provides a more proximal representation of the CNS environment but requires invasive sampling [187]. Plasma and serum are readily accessible biofluids for lipidomic analysis, but their lipid profiles reflect contributions from multiple circulating components, including lipoproteins and extracellular vesicles, and may be influenced by peripheral metabolism, diet, medications, and systemic disease [188,189]. Extracellular vesicles may provide additional information about their cellular sources and molecular cargo. However, reliable EV-associated lipid profiling requires careful isolation and characterization to minimize contamination by lipoproteins and other plasma components, alongside improved analytical standardization and reproducibility [190,191,192]. Therefore, lipid biomarkers identified in different biofluids require specimen-specific validation rather than direct interchangeability. Biofluid-based lipid biomarkers derived from CSF, plasma, and exosomes have been identified as minimally invasive markers to monitor lipid dysregulation in neurodegenerative diseases. Particularly, CSF lipid profiles are valuable due to their close proximity to the CNS and show potential for detecting disease-associated changes in phospholipids, sphingolipids, and sterol metabolites [187,193]. Plasma lipidomics has also shown potential for identifying peripheral lipid signatures associated with neurodegenerative disease pathology, allowing for high-throughput screening and longitudinal monitoring [188]. In addition, exosome-derived lipids could be utilized as a promising avenue for biomarker discovery, since these extracellular vesicles carry lipid constituents that are linked to the cellular origin and disease state [190]. Together, these biofluid-based lipid signatures provide a translational bridge between experimental models and clinical diagnostics, enabling early detection and disease stratification. The ability to perform plasma lipidomic analysis from the same animal models used for tissue lipidomics and transcriptomics further enhances the reliability of identified molecular correlates and biomarkers, bridging the gap between systemic and localized lipid alterations in disease [189]. In CSF, disease-associated markers have been consistently identified, including ceramides, sphingomyelins, sulfatides, phosphatidylcholines, and oxidized phospholipids, many of which represent membrane remodeling and myelin integrity changes in neurodegenerative diseases [187,193]. Plasma lipidomics frequently identifies changes in glycerophospholipids, lysophosphatidylcholines, triglycerides, cholesterol esters, and long-chain polyunsaturated fatty acids, which provide valuable peripheral indicators of systemic lipid dysregulation associated with neurological decline [106,188]. Moreover, exosome-derived lipid profiles contain more sphingolipids, ceramides, phosphatidylserines, and cholesterol-related species, resulting from their neuronal and glial origin, which facilitates early detection of molecular changes associated with synaptic dysfunction and neuroinflammation [189,190,191]. Together, these lipid classes collectively reflect distinct parameters of neuronal membrane turnover, myelin degradation, and neuroinflammatory signaling, further highlighting the relevance of these lipids as fluid-based diagnostic markers.

A published CSF metabolomic and lipidomic study illustrates both the potential and the limitations of lipid-based biomarker discovery. The study included 85 healthy controls, 57 participants with AD, and 56 participants with PD. The lipid-profile classifier achieved an area under the receiver operating characteristic curve (AUC) of 0.75 for distinguishing AD from controls, as reported in the study abstract [192]. However, this finding should be interpreted as discovery-stage evidence rather than proof of a clinically validated diagnostic test. AUC alone does not establish a clinically useful diagnostic threshold, sensitivity, specificity, or predictive value. These measures should be reported only when available from the original study, and independent validation in an appropriate external cohort is necessary before clinical application.

### 6.2. Clinical Applications of Lipid Biomarkers in Neurodegeneration

A statistically significant difference in lipid abundance between patients and controls is not sufficient to establish a clinically useful biomarker. Candidate lipid signatures should be evaluated using ROC analysis, AUC, sensitivity, specificity, confidence intervals, and predictive values where appropriate [125,126,128]. Cohort size, disease stage, specimen type, comparator groups, analytical platform, and the method used to select diagnostic thresholds should also be reported. Independent replication in an external cohort is particularly important because lipidomic signatures can be influenced by biological, pre-analytical, and analytical variability [127,193]. Where a primary study does not report a diagnostic metric or independent validation, this limitation should be stated rather than inferring the missing value. Machine-learning methods may help integrate lipidomic, metabolomic, and clinical data for biomarker discovery; however, their incremental predictive value and generalizability require independent validation [194]. At present, many proposed lipid biomarkers should be considered exploratory until their reproducibility, incremental diagnostic value, and clinical utility have been established. Alterations in ceramide metabolism have been reported in AD and provide a rationale for further investigation of sphingolipid pathways [195,196]. However, the direction and magnitude of these changes, their diagnostic performance, and their specificity for individual neurodegenerative diseases require further validation. Accordingly, ceramide and sphingosine-1-phosphate pathways should be described as potential avenues for biomarker development and therapeutic investigation, rather than established clinical diagnostic targets [195,196]. In addition, future studies should determine whether lipidomic signatures improve diagnostic or prognostic performance beyond established clinical assessments and routinely measured biomarkers. This requires comparison with appropriate clinical models, assessment of incremental predictive value, and evaluation of model calibration and clinical applicability in the intended population. Longitudinal studies may also help establish whether lipid changes precede clinical progression, reflect disease severity, or arise as secondary consequences of neurodegeneration. Ultimately, progress toward clinical implementation will depend on reproducible analytical workflows, adequately powered independent validation cohorts, and evidence that lipidomic measurements provide meaningful information for diagnosis, prognosis, or patient stratification.

### 6.3. Technological Advancements in Lipidomics

Recent advancements in mass spectrometry, such as ultra-high-resolution instruments and ion mobility separation, have led to precise identification and quantification of a wide range of lipid molecules [185]. Single-cell and spatial metabolomics enable the mapping of metabolites in individual cells by addressing the challenge of limited sample availability in precious clinical specimens [197,198,199]. For example, advanced mass spectrometry imaging techniques can provide insights into altered lipid metrics involved in brain function and response to disease such as single-cell level [185,187]. The recent development of high-throughput lipidomics methods such as automated sample preparation and analysis platforms further accelerates the research by allowing the analysis of large sample sets in an efficient and reliable manner [185]. This enables the comprehensive mapping of lipid profiles in tissues containing millions of pixels and in large single-cell populations, providing unprecedented detail of region-specific lipid contents and cell-specific localizations [197]. These advanced methods facilitate high-throughput identification and quantification of lipids, as well as understanding of complex interactions between lipid species and other biological systems [198]. Reliable lipidomic analysis also requires appropriate internal standards, quality-control procedures, normalization, control of batch effects, accurate lipid annotation, and consideration of isomeric species. Targeted and untargeted approaches provide complementary information, while advances in ion mobility, spatial lipidomics, and single-cell lipidomics may improve molecular and cellular resolution.

The development of publicly accessible lipid databases and advanced ionization techniques (electrospray ionization and MALDI), and of mass spectrometry techniques (LC–MS/MS) and imaging allows the detailed characterization and spatial localization of lipid molecules within brain tissues [186,199]. Moreover, comprehensive lipidomic analysis using HPLC or UPLC and electron spray ionization is now possible with high degree of selectivity, specificity, and accuracy that was not previously thought to be possible [185]. These approaches allow for the quantification of known lipid species, but also can be used to discover novel lipids, which is crucial for identification of novel biomarkers and therapeutic targets [200].

#### Multi-Omics Integration

Multi-omics integration can connect genetic variation, lipid metabolic pathways, and protein-level changes within a common analytical framework [201,202]. Genomics identifies disease-associated variants in genes involved in lipid synthesis, transport, and degradation; these findings can be linked to lipidomic measurements to investigate whether genetic variation is associated with specific lipid species or lipid ratios. For example, variants affecting GBA1 can be investigated alongside glycosphingolipid measurements to examine relationships between glucocerebrosidase dysfunction and lipid metabolism [88,89]. Proteomics adds information about the abundance of lipid-metabolizing enzymes, transporters, apolipoproteins, and proteins involved in lysosomal function. Integration can be performed through pathway mapping, correlation analysis, multivariate statistical models, or network-based approaches to identify relationships among genetic variants, proteins, and lipid species [53,201,202]. Studies integrating lipidomic or metabolomic profiles with other molecular datasets illustrate the potential of these approaches to identify disease-associated pathways, including alterations in ceramide and sphingomyelin metabolism [113]. Importantly, correlation across omics layers does not establish causality. Findings should be supported by appropriate statistical control, correction for multiple testing, replication in independent datasets, and, where possible, functional validation. Differences in sample size, measurement platforms, missing data, and tissue or biofluid origin must also be considered when interpreting integrated signatures. Several studies illustrate the application of multi-omics approaches to investigate lipid-related pathways in neurodegenerative disease Table 5.

### 6.4. Personalized Medicine Approaches

The integration of lipidomic profiling may eventually support patient stratification by identifying molecular subtypes, disease-associated signatures, or treatment-response patterns. However, lipidomics-guided personalized treatment remains investigational. Clinical implementation will require analytical standardization, reproducible biomarkers, prospective validation, clinically meaningful thresholds, integration with genetic and clinical variables, and evidence that lipidomic-guided decisions improve patient outcomes. Such lipid-based interventions could potentially improve treatment efficacy and reduce adverse effects by enabling more precise and personalized patient care. However, comprehensive lipid profiling may have the potential to enhance diagnostic accuracy and optimize therapeutic strategies, ultimately leading to improved clinical outcomes [185,203].

### 6.5. Biomarkers and Diagnostic Applications

An important limitation of lipidomic biomarker development is that promising performance in an initial study may not be reproduced in independent cohorts. For example, a previously reported 10-lipid plasma panel for predicting progression from cognitively normal status to AD or amnestic mild cognitive impairment showed an area under the receiver operating characteristic curve (AUC) of 0.92 in a small initial cohort of 28 participants. However, a subsequent study failed to reproduce this performance in two independent cohorts: the AUC was 0.642 in the Baltimore Longitudinal Study of Aging cohort (n = 93) and 0.395 in the Age, Gene/Environment Susceptibility-Reykjavik Study cohort (n = 100) [204]. This discrepancy illustrates why promising discovery-stage results should not be interpreted as evidence of clinical validity. Lipidomic biomarker measurements are influenced by pre-analytical and biological variability, which can limit reproducibility across studies. Pre-analytical factors include collection-tube type and anticoagulant, fasting status, time from collection to processing, centrifugation conditions, haemolysis or blood contamination, storage temperature and duration, freeze–thaw cycles, and lipid extraction procedures. These factors can affect measured lipid abundance and introduce systematic differences between samples. Biological variation also arises from age, sex, body mass index, diet, diabetes, dyslipidemia, medication use, including statins and disease stage. In CSF studies, collection and processing procedures, blood contamination, and differences in participant characteristics require particular attention. In blood-based studies, peripheral metabolic influences may obscure or modify lipid changes associated with the central nervous system. Differences in cohort characteristics, outcome definitions, analytical platforms, lipid annotation, normalization, and statistical modeling may further contribute to inconsistent findings across studies. Reliable lipidomic analysis therefore requires standardized collection and processing protocols, appropriate internal standards, pooled quality-control samples, sample randomization across analytical batches, and transparent reporting of normalization, missing-data handling, and batch correction [1,185,193]. Relevant clinical covariates should be documented and appropriately adjusted for, and sensitivity analyses should assess whether candidate signatures remain stable after accounting for major confounders. Diagnostic models should also be evaluated using appropriate performance measures, including AUC with confidence intervals, sensitivity, and specificity, where available. Ultimately, independent validation in sufficiently large and diverse cohorts is essential to establish reproducibility and determine whether a lipidomic signature has clinical utility.

## 7. Conclusions and Future Perspectives

This review highlighted that, historically, lipids are the most abundant biomolecules in the human brain and were the least explored area of the brain. It is now well understood that lipids are not just structural components of membrane for fluidity and energy storage but also are key regulators of neuronal function, synaptic activity and signal transduction processes. However, emerging evidence reveals that dysregulated lipid metabolism is not merely a byproduct of neurodegeneration but a major contributor to disease progression. Alterations in lipid homeostasis can compromise neuronal integrity, synaptic function, and promote neuroinflammatory responses, contributing to the pathogenesis of neurodegenerative diseases such as AD and PD. Recent advancements in analytical techniques, including enhanced analytical sensitivity, resolution and scan speed, enable the accurate characterization of lipid signatures in biological fluids and tissues. The integration of lipidomics with genomics, metabolomics and proteomics through a multi-omics approach will enable researchers to finally differentiate lipid alterations from disease-associated changes. The understanding of these changes is expected to provide mechanistic insights into disease pathogenesis and could facilitate the identification of robust diagnostic and prognostic biomarkers. Furthermore, understanding of the lipid–protein interactions could open new therapeutic avenues. Certain lipid molecules and membrane-active compounds have the ability to remove toxic misfolded oligomers from cell membranes and stabilize neuronal homeostasis. Current drug candidates are already being designed to target lipid-binding cavities within pathogenic protein fibrils, such as α-synuclein, to prevent the progression of neurodegeneration. This review highlights how establishing a comprehensive lipidome map across diverse brain regions and cell types is essential for uncovering the functional roles of lipids in the CNS. Collectively, this comprehensive approach is essential for identifying robust biomarkers and developing personalized, disease-specific interventions such as targeted nutritional modification or pharmacological agents that maintain brain function during the physiological aging process. However, important challenges remain, including analytical standardization, lipid annotation, biological and pre-analytical variability, limited independent validation of candidate biomarkers, and uncertainty regarding causality. Further studies using larger and diverse cohorts, standardized workflows, longitudinal designs, and integrated multi-omics approaches are required to establish the clinical utility of lipidomic signatures.

## Figures and Tables

**Table 1:** Comparison of targeted and untargeted lipidomics approaches for neurodegenerative disease.

Feature	Targeted Lipidomics	Untargeted Lipidomics
Primary purpose	Quantification of predefined lipid species	Broad profiling and discovery of candidate lipid features
Best for	Testing a defined hypothesis or validating a candidate biomarker	Identifying previously unanticipated lipid alterations
Typical analytical approach	Targeted LC–MS/MS, often using MRM	Broad-spectrum MS profiling, often using high-resolution MS
Quantification	Can support absolute quantification with appropriate calibration and standards	Often relative or semi-quantitative unless quantitative validation is performed
Advantage	Focused measurement and method optimization	Broader discovery potential
Limitation	Restricted to predefined analytes	More complex annotation, missing-data handling, and multiple-testing challenges

**Table 2:** Integrative Lipidomic and therapeutic strategies in neurodegeneration in various in vitro cell culture models.

Category of Drug	Drug Name	In Vitro Model	Effect on Lipid/Membrane Context	Ref.
Aminosterols	Trodusquemine	Protein oligomer–membrane assays; cellular toxicity experiments	Displaces misfolded protein oligomers from model membranes and reduces membrane-associated cytotoxicity	[160,161]
Oligomer Modulators	Anle138b	α-synuclein and prion protein aggregation assays; membrane-related mechanistic assays where specifically reported	Modulates pathological protein aggregation. Do not claim prevention of membrane poration unless the cited experiment directly measured membrane permeabilization or damage	[162,163]
Gut Metabolites	Urolithin A	LPS-stimulated BV2 microglia; APPSwe-transfected ReNcell VM human neural cells	Reduced nitrite, TNF-α and IL-6; increased SIRT1 and autophagy-related activity; improved cell viability in the neural-cell model. These results support anti-inflammatory and autophagy effects, not direct restoration of mitochondrial lipid composition	[164,165]
GLP-1 Receptor Agonists	Semaglutide	LPS + ATP-stimulated BV2 microglia	Attenuated inflammatory ferroptosis along with the inhibition of NLRP3 inflammasome activation and modulation of NF-κB and Nrf2/HO-1/xCT/GPX4 signaling	[166,167]
Aminosterols	Squalamine	Synthetic anionic lipid vesicles	Competitively displaces synuclein from the lipid-membrane surface, inhibiting the early stages of protein aggregation	[161]
Antioxidant	α-Lipoic Acid	Neural tissue and neuronal injury models, depending on the cited experiment	Potently inhibits lipid peroxidation and restores redox balance in mitochondrial membranes	[71]
Exosome Modulators	BDE-targeted agents	LC-MS profiling of isolated exosomal vesicles	Identifies and modulates disease-specific lipid and phosphoproteomic signatures within exosomal membranes	[168,169]

**Table 3:** The emerging therapeutic approaches centered on lipid homeostasis and experimentally validated in various in vivo models.

Category of Drug	Drug Name	In Vivo Model	Effect on Lipid Context	Ref.
GLP-1 Receptor Agonists	Semaglutide	APP/PS1 transgenic mice	Attenuated neuroinflammation by suppressing ferroptosis, lipid peroxidation and NLRP3 inflammasome activation in microglia	[166,171]
Oligomer Modulators	Anle138b	Rotenone-induced and MI2/C57Bl/6S PD mice	Inhibits protein aggregation that typically binds to and pores anionic lipids in neuronal membranes	[162,163]
Antioxidants	α-Lipoic Acid	AlCl3 and d-galactose-induced AD rats	Potently inhibits lipid peroxidation; restores mitochondrial redox balance and membrane stability	[71]
Gut Metabolites	Urolithin A	Cadmium-induced cognitive deficit mice	Attenuated cognitive deficits and NLRP3-mediated pyroptosis through regulation of aryl hydrocarbon receptor signaling	[172]
Incretins	Liraglutide	3xTg and 5xFAD mice	Protects cerebral vasculature lipids and reduces amyloid-induced lipid-membrane damage	[173]
Exosome Modulators	BDE-targeted agents	Human clinical biosignatures and preclinical mouse model of PD	Normalizes the phosphoproteomic and lipid-vesicle signatures within brain-derived; downregulation of exosomal miR-137 alleviates oxidative stress injury in PD by upregulating OXR1	[174]

**Table 4:** Clinically evaluated therapeutics influencing lipid-membrane or lipid–protein-associated mechanisms in neurodegeneration.

Category of Drug	Drug Name	Clinical Trial Subjects	Clinical Trial Registration Number	Main Findings Relevant to Lipid/Membrane Biology	Clinical Status and Interpretation	Status	Ref.
Lipid–protein interaction/Misfolding Inhibitor	Minzasolmin (UCB0599)	Adults with early-stage Parkinson’s disease	NCT04658186 (ORCHESTRA); NCT04875962	Designed to inhibit α-synuclein misfolding and aggregation. The proposed lipid-membrane mechanism remains a mechanistic rationale rather than a demonstrated clinical benefit	Phase 2a ORCHESTRA did not meet its primary or secondary clinical endpoints. The program was discontinued; it should not be described as an ongoing efficacy trial	Completed	[176]
GLP-1 RA (indirect metabolic comparator)	Semaglutide	Early Alzheimer’s patients (amyloid-positive, MCI)	NCT04777396; (EVOKE); NCT04777409 (EVOKE+)	Investigated for broader metabolic and neuroinflammatory effects. A direct lipid-targeted or blood–brain barrier lipid-restorative mechanism was not established by these trials	Phase 3 trials did not demonstrate statistically significant slowing of clinical progression. Retained as an indirect metabolic comparator, not a validated lipid-targeted therapy	Discontinued	[175,181]
Aminosterol/membrane-active agent	ENT-01	150 patients with Parkinson’s and constipation	NCT03781791	Studied for membrane interactions and displacement of α-synuclein from membranes. Clinical evidence primarily concerns gastrointestinal outcomes.	Phase 2b study completed. Constipation and exploratory neurological outcomes should be reported separately; the findings do not establish disease modification in Parkinson’s disease	Completed	[182]

**Table 5:** Examples of multi-omics integration relevant to lipid biology and neurodegenerative disease research.

Data Integration or Focus	Main Contribution	Limitations	Ref.
Plasma and CSF metabolomics integrated with CSF proteomics	Examined associations between lipid mediators and CNS-related vascular and energy metabolism	Associations do not establish causal relationships	[53]
Multi-omics analysis of ceramide/sphingomyelin pathways in Alzheimer’s disease (AD)	Identified lipid-related pathways for further investigation as potential therapeutic targets	Pathway identification does not establish clinical efficacy	[113]
Integrative multi-omics and systems bioinformatics	Described approaches for connecting molecular datasets in translational neuroscience	Broad methodological perspective; not a disease-specific validation study	[201]
Network-based omics approaches in neurodegenerative diseases	Discussed network approaches for examining molecular relationships in disease	Conclusions depend on dataset quality, study design, and independent validation	[202]

## Data Availability

No new data were created or analyzed in this study. Data sharing is not applicable to this article.

## Abbreviations

The following abbreviations are used in this manuscript:

AA	Arachidonic Acid
ABCA1	ATP-binding cassette subfamily A member 1
ABCG1	ATP-binding cassette subfamily G member 1
ACSL1	Acyl-CoA synthetase long-chain family member 1
AD	Alzheimer’s disease
Aβ	Amyloid-beta
AI	Artificial intelligence
ALS	Amyotrophic lateral sclerosis
APOE	Apolipoprotein E
APOE4	Apolipoprotein E ε4
APP	Amyloid precursor protein
AUC	Area under the curve
BBB	Blood–brain barrier
CNS	Central nervous system
CSF	Cerebrospinal fluid
DGAT1/2	Diacylglycerol O-acyltransferase 1/2
DHA	Docosahexaenoic acid
EVs	Extracellular vesicles
GBA1	Glucosylceramidase beta 1
GlcCer	Glucosylceramide
GlcSph	Glucosylsphingosine
GLP-1	Glucagon-like peptide-1
HD	Huntington’s disease
HMG-CoA	3-Hydroxy-3-methylglutaryl coenzyme A
HPLC	High-performance liquid chromatography
IL-1β	Interleukin-1 beta
LC–MS/MS	Liquid chromatography–tandem mass spectrometry
MALDI	Matrix-assisted laser desorption/ionization
MCI	Mild cognitive impairment
ML	Machine learning
mTOR	Mechanistic target of rapamycin
MSA	Multiple system atrophy
NPV	Negative predictive value
OXR1	Oxidation resistance 1
PC	Phosphatidylcholine
PD	Parkinson’s disease
PE	Phosphatidylethanolamine
PERK	Protein kinase R-like endoplasmic reticulum kinase
PLIN2/3	Perilipin 2/3
PP2A	Protein phosphatase 2A
PPV	Positive predictive value
PS	Phosphatidylserine
PSP	Progressive supranuclear palsy
PUFA	Polyunsaturated fatty acid
RA	Receptor agonist
ROC	Receiver operating characteristic
S1P	Sphingosine-1-phosphate
SIRT1	Sirtuin 1
SMase	Sphingomyelinase
SNARE	Soluble N-ethylmaleimide-sensitive factor attachment protein receptor
SMPDE	Sphingomyelin phosphodiesterase
TNF-α	Tumor necrosis factor-alpha
UPLC	Ultra-performance liquid chromatography
VAMP-2	Vesicle-associated membrane protein 2
4-HHE	4-Hydroxy-2-hexenal
4-HNE	4-Hydroxynonenal
α-syn	Alpha-synuclein
eIF2α	Eukaryotic initiation factor 2 alpha
miR-137	MicroRNA-137
